# Conceptualising wellbeing among health-care workers during the Covid-19 pandemic

**DOI:** 10.1177/13634593241279206

**Published:** 2024-10-06

**Authors:** Judith McHugh, Paul Trotman, Helen D. Nicholson, Kelby Smith-Han

**Affiliations:** University of Otago, New Zealand; Te Whatu Ora - Southern, New Zealand; University of Otago, New Zealand; University of Western Australia, Australia

**Keywords:** Capability Approach, content analysis, Covid-19 pandemic, healthcare professional, wellbeing

## Abstract

Since 2020 health workers everywhere have been challenged by the ongoing ramifications of the Covid-19 pandemic. This virus impacted all aspects of life but health-related workplaces particularly, were transformed virtually overnight. Demands were heightened and customary supports came under pressure presenting a huge crisis for health systems. The goal of this study was to explore how this catastrophic pandemic event impacted the wellbeing of healthcare professionals (HCPs) working through this time. Interviews with 57 HCPs from multiple countries and specialty areas were explored utilising inductive content analysis (ICA). Resulting data were then categorised into themes and deductively analysed utilising a method informed by Capability Theory. These were secondary data as the interviews were part of a larger set collected primarily for the purpose of a documentary being made about this experience. This study found that illbeing experiences were prevalent among HCPs. However, significant sources of wellbeing were also evident, and were instrumental in maintaining HCP resilience. Wellbeing was enhanced when HCPs experienced a small number of key *capabilities* that enabled a broad range of *functionings.* The *capabilities* were for (a) participation in positive relationships, (b) a sense of identity, purpose, meaning and value in relation to one’s work and (c) ability to provide an appropriate level of medical treatment, care and other role related support. These c*apabilities* were central to HCP wellbeing irrespective of the individual’s location and specialty area, however the ability to realise these *capabilities* in desired *functionings* was differentially impacted by each individual’s unique circumstances.

## Introduction

The Covid-19 pandemic heightened concern for HCP wellbeing globally. This was evident in the large number of studies produced describing the mental health impacts on HCPs (Alkandari et al., 2021; Billings et al., 2021; Creese et al., 2021; Cubitt et al., 2021; Czepiel et al., 2022; Khajuria et al., 2021; Lai et al., 2020; Pappa et al., 2020). Hennein and Lowe (2020) for example, utilised multiple measures of individuals’ mental ill-health to quantify wellbeing impacts in relation to a range of social and ecological factors. Of 1132 participants 14% were identified as having experienced major depression, 15.8% with generalised anxiety disorder, 23.1% probable post-traumatic stress disorder and 42.6% probable alcohol use disorder.

Wellbeing however, is a broader concept and more contextual than a dysfunction or an illness-based analysis implies (Watson et al., 2023). It describes a positive state of being, related to human flourishing and being grounded in one’s whole of life experience (Ryan and Deci, 2001; Ryff and Singer, 2008; Seligman, 2002; Simons and Baldwin, 2021). It therefore needs to be understood multi-dimensionally. Numerous attempts have been made to identify key dimensions, components, or determinants of wellbeing but these largely remain focussed on individual psychology and have limited applicability across different cultures.

Currently, the positive psychology approach to wellbeing is popular across western societies (Seligman and Csikszentmihalyi, 2000). This emphasises individual capability and personal responsibility for one’s own wellbeing, taking relatively little account of external factors and organisational structures that may be impacting on a person. This focus sits well alongside the prevailing neoliberal philosophies that underpin western-style economies, and that prioritise individual responsibility (Hammoudeh, 2021). It is a perspective frequently criticised by those utilising more sociological approaches which take greater account of a person’s subjectivity and situatedness in determining wellbeing (Atkinson, 2013; Atkinson et al., 2020; Searle et al., 2021). Other wellbeing perspectives that have been influential historically include Basic Needs theory (Adie et al., 2008; Tay and Diener, 2011), Economic approaches (Bergheim et al., 2006; Stiglitz, 2009), Utilitarianism (Mulgan, 2014) and Subjective Wellbeing (SWB; Diener and Ryan, 2009).

### Capability Approach

In response to perceived shortcomings in these approaches, Amartya Sen developed *Capability Theory* (1993) which argues that a person’s wellbeing is contingent upon what they are *able to be, and do*, in relation to their desired or valued outcomes. When utilising a Capability Approach (CA) the focus remains on the individual, however it crucially acknowledges external influences and constraints on capability and thus wellbeing. A CA is an agency-based model focussing on what people can *actually* achieve in their lives (Sen, 1993). This shifts wellbeing conversations beyond concentrating on the resources people have, to what they are actually, in reality, able to be and do with those resources.

A CA focuses on the concepts of Achievement and Freedom (Robeyns, 2005). Achievement *(*i.e. *Functionings)* is what we manage to accomplish and ‘Freedom’ *(i.e.Capability) is* the ‘*real*’ opportunity we have to accomplish what we value (Crocker and Robeyns, 2009). Sen understood that the key to individual wellbeing was having the *freedom to choose* how to utilise available resources to achieve a desired goal or quality of life (Sen, 1985). A CA acknowledges that different people will make different choices regarding this and that these choices will be significantly influenced by structural aspects, such as cultural, socio-economic and institutional factors, as well as the personal (Robeyns, 2005). Importantly personal agency achievements are not always connected to wellbeing, for example, when older or immune-compromised HCPs choose to continue working despite their elevated risk.

In a CA, factors determining the achievability of desired *functionings* are called *conversion factors* (Alkire, 2008). Each person is subject to a unique set of *conversion factors* that govern the degree to which they can utilise opportunity to achieve desired *functioning* (Bifulco et al., 2010). There are personal *conversion factors* pertaining to the individual such as health, knowledge, fitness, or strength; there are social *conversion factors* that include policies, practices, hierarchies, power relations related to class, gender or race, family structures and there are environmental *conversion factors* related to the physical or built environment one lives or works in (Robeyns, 2017). Using a CA the key question becomes what individuals are actually able to be and do in their lives given their individual contexts and circumstances and how this ultimately impacts their wellbeing.

### Covid-19

In 2020 health systems around the world were thrown into turmoil with the emergence and rapid spread of the Covid-19 virus. As countries struggled to mobilise resources quickly and effectively to navigate this upheaval, many HCPs found themselves at the frontline in a crisis, the like of which most had never before experienced (Wong et al., 2021). Enormous strain was placed on many HCPs generating serious wellbeing challenges (Digby et al., 2021; Lai et al., 2020; Vanhaecht et al., 2021). The most commonly reported concerns were anxiety, depression, grief, fear, anger, sleep disturbances and post-traumatic stress (Bozdağ and Ergün, 2021; Rossi et al., 2020). Frontline HCPs, especially nurses, in covid-positive areas were more likely to report psychological disturbances compared to those working in non-covid areas (Di Mattei et al., 2021).

Ironically though, there was also clear evidence of positive wellbeing experiences occurring for HCPs. These included experiences of teamwork (Anjara et al., 2021; Vindrola-Padros et al., 2020), mutual support (Vera San Juan et al., 2021), deep sense of meaning and purpose (Dalle Ave and Sulmasy, 2021), and job satisfaction (Savitsky et al., 2021; Yu et al., 2020). It appears that such experiences help to ameliorate some of the negative effects of the pandemic.

This study highlights HCP’s experiences of positive wellbeing during this crisis and how this impacted their resilience. A CA was used to explore this because of its capacity to address multiple dimensions of wellbeing (Robeyns, 2005). It focuses also on the difference between substantive freedoms (*capabilities*) and outcomes (achieved *functionings*) which, given the impact Covid19 was having on HCPs freedom and ability to practice, seemed appropriate.

## Methods

### Methodology

An abductive content analysis (ACA) methodology was utilised to facilitate a broad-based description of the impacts of the pandemic on HCP’s wellbeing (Graneheim et al., 2017). As well as enabling descriptive content analysis this methodology has the capacity for latent and interpretive analysis which, while more distant from the text remains close to the participants’ lived experience (Graneheim and Lundman, 2004). ACA involves moving between both inductive and deductive processes for a more complete understanding of the phenomena (Graneheim and Lundman, 2004). The deductive phase of the analysis was informed by Capability Theory (Sen, 1993).

### Data collection

Between April and August 2020 an international group of HCPs working across different disciplines and specialty areas were interviewed about their experiences working through the early phases of the pandemic. This was for a documentary being made by the second author about this experience (Trotman, 2021). The initial sample group was identified by PT (second author and longstanding research collaborator of HN (third author)), using key informants (Marshall, 1996) from personal networks and social media posts combined with a snowball sampling approach (Sedgwick, 2013). This process yielded an opportunistic sample group of 112 HCP’s from across 12 different countries who represented multiple disciplines and specialities. The interviews were semi-structured and conducted by a single interviewer (PT) using online technology. The semi-structured interview guide is presented in Supplemental Material. Ethical approval required that inclusion in our sample be determined by our ability to obtain further specific written consent from the HCPs, granting us access to their data for this study. All original interviewees were approached and 57 written consents were obtained. The HCPs included in our study were from a range of locations and specialty areas. In order to maximise participant numbers no attempt was made to stratify the sample.

### Data analysis

Audio and transcript recordings were analysed qualitatively using Abductive Content Analysis (ACA; Graneheim et al., 2017). Data were stored securely and managed electronically utilising the ATLAS.ti (version 9.1.3).

**Figure 1.**
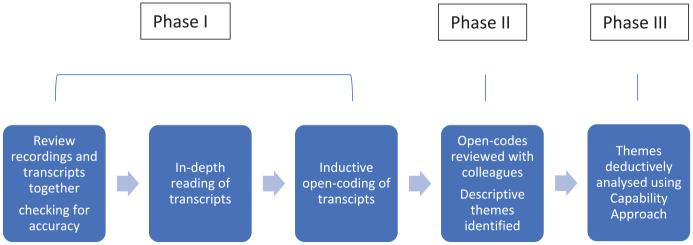
Data analysis.

Figure 1 outlines the data analysis process.

**Phase I:** Recordings and transcripts were reviewed to gain overall impressions of the data and to check for accuracy. The first author (JM) immersed herself in the data by first listening to all the recordings. In-depth reading of the transcripts followed. Interviews were inductively open-coded for ‘wellbeing impacts’ yielding 247 codes. Wellbeing impacts were identified either explicitly by the subjects themselves, or through intuitive, empathic processing by the first author utilising contextual information, content and tone of voice. Unfortunately, there was no opportunity to obtain further clarification from participants.

**Phase II:** Categories and concepts were developed from the raw codes maintaining sensitivity to individual content and context (Harwood and Garry, 2003; Kyngäs, 2020). This enabled greater flexibility dealing with meanings and intentions and identifying critical processes (Harwood and Garry, 2003). An iterative process was implemented on the codes and data and initial themes were identified (Kekeya, 2016). The initial themes were discussed by JM, HN and KS (fourth author). The themes were then refined using the constant comparison method by these same authors (Smulowitz, 2017). The constant comparison method is a tool that originated from Grounded Theory but is not restricted to Grounded Theory itself. The constant comparative method is a general intellectual tool used in qualitative analysis involving comparing and contrasting, and can be used in many areas including the process of coding and theme development (Boeije, 2002; Fram, 2013).

**Phase III:** The codes and themes developed in Phases I and II were interpreted deductively utilising a CA (Robeyns, 2017). Key principles of Capability Theory (Robeyns, 2017) were applied to the results of the thematic analysis by JM. This process was discussed and refined with HN and KS.

## Findings

### Demographic summary

Tables 1 and 2 summarise the characteristics of study participants. Twenty-seven of the males were physicians compared to 14 of the females.

**Table 1.** Geographic location at time of interview.

Location at interview	Male (*n* = 33)	Female (*n* = 24)	Total (*n* = 57)
Australia	**4**	**3**	**7**
France	**-**	**2**	**2**
Italy	**1**	**-**	**1**
Japan	**1**	**-**	**1**
New Zealand	**13**	**6**	**19**
Scotland	**4**	**-**	**4**
USA	**6**	**9**	**15**
UK	**4**	**3**	**7**
Spain	**-**	**1**	**1**

**Table 2.** Work role/area.

Role	Male	Female	Total
Anaesthetist	3	3	6
Emergency Med	4	4	8
General Practitioner/Family Med	8	6	14
House officer/Registrar	1	2	3
ICU/Crisis Team	3	4	7
Infection control /internal medicine	2	1	2
Managerial role/clinical co-ordinator	4	3	7
Medical Educator/Appraiser	1	1	2
Medical Supplies	1	-	1
Palliative Care	2	4	6
Paramedic	2	1	3
Consultant/specialist	8	1	9
Psychiatry	1	2	3

Nine participants identified as a nurse or nurse-practitioner. The group included one laboratory scientist and one person operating a company providing medical supplies. HCPs often had multiple roles. The detail In Table 2 illustrates their diverse involvements. As a group they were highly articulate and deeply reflective about their experiences and responses to working through the pandemic crisis.

### Inductive analysis

Supplemental Table 1 shows how inductive thematic analysis was utilised to capture aspects of both HCP illbeing and wellbeing. The first identified theme describes the illbeing experiences most commonly reported by interviewees. The remaining three themes reveal the most frequently reported sources of HCP wellbeing.

### Illbeing

Experiences of illbeing and distress were prevalent and significant among HCP’s. Two important exacerbating factors were (a) dealing with the constant, significant, widespread changes that occurred in a short space of time; and (b) dealing with the tsunami of information that descended upon the health workplace. Demands changed sometimes by the hour, processes and protocols were altered daily, staff were being moved around and there were major adaptations to the workplace. Understandably this aggravated work stress, fatigue, and anxiety.

*Things that my agency was doing one day, within a few days, we’re doing something different. Today, this is the PPE you’re supposed to wear but by tomorrow (it’s) something different. . . there are multiple orders out and pieces of paper. Every day to come to work, you had four or five pieces of paper you had to study. . .ok, so this is what we’re doing today. At times it felt like (I was) drinking from a fire hose. Cause it’s just too much information. (Paramedic, USA)*

The early phases of the response were particularly difficult for HCPs emotionally and psychologically, as whole systems and everyone in them struggled to adjust to dealing with covid. Morbidity and mortality rates were frighteningly high and unpredictable and the threat to self and loved ones was a very present and real danger. Those who coped best were able to fall back on their training and focus on the work at hand.

*. . .the first time I saw a patient present with severe hypoxia, with oxygen saturations in the sixties, and the need to emergently intubate that patient. That was a very scary time, but that’s where, going into professional, trained autopilot was important. . .the anxiety levels were the most profound that I have seen in my entire career. But the culture of emergency medicine in particular, has really shone in this time, the sense of team, of flexibility, of doing the best we can with what we have got. All of these things have shown themselves to be true, and seeing people step up and achieve, incredibly impressive things, in such a short space of time. . . (Emergency Medicine Physician, Melbourne)*

### Deductive analysis

#### Capabilities

In this study three *Capabilities* clearly emerged as being particularly important to HCPs’ wellbeing. These were having the capability for (a) participation in positive relationships (especially collegial ones), (b) a strong sense of identity, purpose, meaning and value in relation to the work at hand and (c) being able to provide an appropriate level of medical treatment, care, and other work role-related support. The boundaries between these capabilities were permeable in that they constantly blended and reinforced each other. Further analysis of what constituted each capability is presented below.

#### Participation in positive relationships

This core capability was central to HCPs wellbeing. Ideally, positive relationships extended across all areas of life including work and colleagues, family and friends, neighbourhood and communities, as people primarily turned to each other for help and support to get through their days. Here we will focus primarily on work-related relationships. Table 3 shows the most frequently reported expressions of positive relationships that supported HCP wellbeing.

**Table 3.** Participation in positive relationships.

Opportunities for participation in positive relationships
**Being part of a team** showing support, assistance, empathy and concern for each other.
**Mutual support** appreciating wider collegial relationships and networks (including international)
**Relational wellbeing** relationships with work, workplace, families, community, and wider political events
**Staff morale, collegiality & respect** reduced power relations, co-operative decision making, increased accommodation and respect for difference, relations with other services
**Wellbeing Support** mutual support & actions that came from outside profession for example, wider industry support (e.g. with technology or PPE.)

Broadly speaking these categories were distinguished by different levels of proximity, with the most important relationships occurring among immediate colleagues. Solidarity, collegiality, camaraderie, teamwork, were the words used to describe the best thing to come out of this experience for HCPs. It is clear from the quotes below how important these relationships were for maintaining morale and wellbeing when experiencing extreme stress.

*. . .(an) incredible sense of camaraderie I have to say, like for the most part, everybody’s just really stepping up . . . the teamwork is really beautiful right now. Its one of the good things that has come out of this. I want to go to work, actually I’m almost excited to go to work, even though it’s so hard and it’s heart-breaking. . . (ICU nurse, USA)*

*I became so humbled by how my faculty performed. They stepped up and stepped in, in ways I can’t even put together in words and that courage and that commitment and compassion that they showed each other too, to not have expectations of each other, but just to be there when they could. I told them it was the fuel in my engine. It’s what kept me going because their behaviour went above and beyond. Those are the things that get you past the fear when, you know, you can make a difference when you have a sense of connection and purpose and the human contact helped too. (Neonatal Specialist, USA)*

Good relationships held everything together irrespective of location or clinical setting. Barriers to communication such as the use of PPE and physical distancing had to be overcome or compensated for quickly in order to maintain collegial relationships and to do the work to hand. Teamwork was the mainstay of successful adaptation and coping through this time. Technology helped, keeping teams connected with innovative use of various devices and computer applications.

When this capability for good relationships could not be actualised the loss of associated functioning caused a kind of grief. For example, in the early response period especially, many HCP’s reported that they struggled to build relationships, and have confidence in their practice, due to the constraints of providing remote care or communicate effectively through the veil of PPE.

As HCPs adjusted to new ways of working their confidence and wellbeing improved. The possibilities of practice in this new environment became more apparent and attractive but individuals still lamented what had been lost.

Increased collaboration across hierarchies, departments, disciplines, institutions, and countries was a beneficial aspect of the pandemic response. Not only did collaboration inform understanding, treatment and care, it also strengthened a wider sense of team, of all pulling together in the same direction, of mutual support and positive regard.

Staff wellbeing was further enhanced when managers and consultants were seen to join frontline workers assisting with providing cares.

*I think you got pretty amazing time with the consultants. They were very much on your level. . .One of the main ones I worked with was a rheumatologist who was a professor. Hadn’t done much clinical medicine in a while and you know, it wasn’t such a seniority. Because it was such a new thing, he felt very much on our level, but had that little bit more experience to guide us. I think it broke down the barriers between specialties quite a lot as well. . . (House-officer, UK)*

In the early phases of the pandemic when fear in the community was running very high HCPs sometimes experienced disconnection, being shunned as people avoided them in the street, neighbours and friends withdrew, or being verbally abused for going to the supermarket in ‘clean’ scrubs. This exacerbated HCP’s sense of isolation and sometimes led to increased anger or sadness.

*I’ve lived in my same building for six years. . . .We’re generally a tight-knit community. . .I’m paramedic, so at the beginning, when the media driven hysteria was really getting pretty bad in New York . . .. No one knew anything except everyone was getting sick and everyone was dying and everything was shut down. I got home to a note that had been slipped under my door that suggested it was irresponsible for me to still be living here that I should move out, not to do my laundry, not to throw away my garbage because I brought Corona in the building. Which kinda knocked me a little bit because this is a building in which I know all of my neighbours. . .And at times when they’ve had issues they come to my door, I never turn anyone away. But now I’m getting turned away. So it was, it was really hard to deal with. (Paramedic, USA)*

#### Identity/purpose/meaning/value in relation to one’s work

The second key capability identified in this study was for ‘Identity, Purpose, Meaning and Value’ in relation to one’s work. A number of different permutations expressing this capability were mentioned, for example playing one’s part, willingness to put oneself at risk, sense of responsibility, pride in workforce, pride in country’s response, feeling valued, dedication to service, personal affirmation/appreciation, being part of something bigger, self as helper or hero.

Table 4 shows the different aspects of this capability most frequently mentioned by interviewees. As with the relationship capability these aspects were not distinct but flowed into and informed each other. Not being able to fulfil this capability posed a serious negative challenge to HCP wellbeing.

**Table 4.** Sense of identity, purpose and meaning and value.

Opportunities to experience Meaning, Purpose and Identity
**Identity – role** (as a doctor, nurse, paramedic, GP, in Palliative care, ICU or ED etc.)
**Sense of purpose** (Having a part to play/ being part of the response/making a contribution)
**Willingness to put self at risk to help**
**Identity – sense of responsibility** (the ‘right’ thing to do, what I signed up for)
**Identity – sense of self** (the deeply personal response to one’s own situation vis-à-vis work**)**

Many of the HCPs in this study spoke of being confronted at some point by questions such as ‘Why are you doing this work?’ ‘Why are you putting yourself through this?’ The answer came back ‘Because that’s what I do, I am a nurse/doctor/paramedic. . .’ ‘This is what I signed up for’ or ‘I do this because I have the skills’ or ‘I want to help people.’ In this study those who had this strong sense of identity with their work appeared to gain strength and resilience from it.

*I didn’t really understand how much I loved people to continue doing what I’m doing. . . I think that we’re all in a position that we had to take a hard look at ourselves and it was by force. This was not something that we chose to do. This was something that we were forced to do. We had to sit there and dig down really deep and understand, ‘Why are you doing this? Why is it that you’re getting up today and putting on a uniform and are you here for the money? Are you here for the glory? Because neither of those things are occurring right now. So, if you’re still here, why are you here?’ (Paramedic, USA)*

Positive identification with one’s work role served to strengthen individuals’ sense of meaning and purpose. As can be seen from the following quote these three factors together proved a powerful contributor to HCP wellbeing and functioning.

*The good news about coming into the hospital every single day and exposing yourself every single day, was it gave me the privilege of a sense of purpose. So the way I balanced the fear was to say, “I’ve got a sense of purpose. . . we have a really important job to try and save lives. And these lives can be saved. So the sense of purpose was important. (Neonatal specialist, USA)*

The negative aspect of this capability occurred when HCPs who identified so much with their work as a vocation experienced failure or futility when treatments failed and people died despite their best efforts.

*Anyone that works in medicine or healthcare where that’s the vocation you’ve chosen in life, you get part of your self-being when you’re able to help. I’ve been a paramedic for a long time and coming to the realisation during the coronavirus pandemic, that it didn’t matter what I was doing. I was not helping these people. There were people that I would get as patients on my ambulance and I sat there and consciously realised that I would likely be the last person they spoke to because they look fine right now, but their oxygen saturation level is 38. . .And I understood inherently that every time I took a set of vital signs and some crazy number popped up, that in the next couple of hours, that person would be on a ventilator, if not dead. And it was a very sobering, a very mentally destroying understanding to come to, that your vocation in life has been rendered completely obsolete. (Paramedic, USA)*

HCPs’ sense of identity and self-worth was often similarly challenged when they were prevented for whatever reason from working with colleagues and contributing to the pandemic response. Decisions around whether and when to put oneself at risk were extremely difficult for some individuals to navigate, especially older HCPs who were encouraged to stay away from work despite feeling healthy and able to work.

*The hardest thing was stepping away from my profession for a period of time. We dedicate a lot of energy into becoming physicians. That was an extremely hard decision for me that week when I decided, ‘okay, I’ve got to make a choice here between continuing to work on my life’s profession or just step away for my family’s benefit for my own benefit, what my grandchildren need, what my children need’. So that was the hardest, actually stepping away and not working to help during a crisis. That’s not usually the way I work. . . it literally felt cowardly. . .I didn’t want to abandon my colleagues . . . they were not abandoning me. They were looking out for me. And that was the hardest part. (Family Medicine Physician, USA)*

The sense of letting others and oneself down, was deeply disturbing and in some cases completely untenable, as expressed by this doctor.

*I think your conscience for the rest of your life would be so disturbed at the abandonment of ethical principles, of patient care and responsibility, professional responsibility. I think your conscience wouldn’t be worth living with if you actually said, ‘There’s no way I’m going in there.’ If somebody’s life depended on it. In other words, if there’s nobody else to do that, and it has to be done and you say, ‘No way, because there’s no PPE,’ I don’t think you could live comfortably with yourself for very long.* (*Respiratory physician, Scotland)*

#### Capability for providing appropriate medical treatment, care and other role-related support

Given the context of this study the capability for work was fundamental, all-encompassing and inextricably linked to everything. Table 5 shows the *functionings* that were fundamental to HCP wellbeing in the workplace. Illbeing was evident in relation to the degree that these could not be experienced.

**Table 5.** Capability for provision of appropriate medical treatment, care and other role-related support.

Opportunities for providing appropriate treatment, care and service
Being able to manage risks effectively
Being able to access and utilise available resources and support
Being able to access the necessary information in a manageable way
Being able to take opportunities for self-care
Having access to supportive management structures
Good leadership

It was clear that being in a position to perform competently and effectively in one’s work role was central to participants’ wellbeing and that this capability was seriously undermined by events and by health systems that were struggling to cope.

## Discussion

The aim of this research was to explore wellbeing among HCPs working through the Covid-19 pandemic. We used a Capability Approach (Sen, 1993) to reflect on this question because of its capacity to encompass an individual’s situatedness, as well as their subjectivity in relation to wellbeing. Study participants worked in different parts of the world and across a range of disciplines and roles. Although this was an opportunistic study of material collected for other purposes, the data obtained revealed much about the wellbeing experiences of healthcare workers at this time.

Despite the variety of individuals and circumstances represented in this study, four wellbeing related themes clearly emerged from the data. The first of these related to the significant illbeing experiences generated for HCPs working during the early months of the pandemic. These findings concur with previous research showing HCPs, especially those working in the front line (Di Mattei et al., 2021), experienced significantly increased symptoms of mental, psychological, emotional, and physical distress (Digby et al., 2021; Hennein and Lowe, 2020; Lai et al., 2020). Conversely, this current study focuses on how HCPs experienced ‘positive’ wellbeing during this stressful time.

The three remaining themes in our study suggest that three core capabilities underpin HCP wellbeing. These were: a) capability for participation in positive relationships, b) for a strong sense of identity, purpose, meaning and value in relation to one’s work and c) ability to provide appropriate medical treatment, care and other role-related support. Irrespective of the location or specialty of HCPs these three capabilities emerged as essential to individual wellbeing. This finding bears some similarity to Ryan and Deci’s Self Determination Theory (SDT; 2017) which identifies three fundamental psychological needs underpinning wellbeing (Ryan, 2009). These were for relatedness, competence, and autonomy. Of these, the first two closely align with our capability findings but the third, that of ‘autonomy’ does not (See Table 6).

**Table 6.** Core Capabilities compared to Basic Psychological Needs.

**Capabilities**	**Basic Psychological Needs** (Ryan and Deci)
Ability to provide appropriate medical treatment, care and other role-related support.	Competence
Participation in positive Relationships	Relatedness
Identity, purpose, meaning and value at work	Autonomy

This study suggests that having the capability to connect with a personal sense of identity, purpose and meaning (in relation to one’s work) preceded the need for autonomy. In a time when usual sources of wellbeing were scarce, being able to express this capability strengthened HCP wellbeing and resilience. Thinking about what one was able to achieve (i.e. autonomy) was secondary to the question of why one wanted to achieve it.

### Conversion factors

*Conversion factors* govern the degree that capabilities can be realised in valued *functionings*.

Much of the research emerging from this pandemic highlighted negative *conversion factors*, especially system related factors that hindered or prevented HCPs from fulfilling their capabilities, for example, poor access to appropriate workspaces and resources. Such factors inevitably resulted in increased stress and potentially significant levels of illbeing. The system-related conversion factors most evident in this study related to information and communication management, resource availability, staff support and leadership.

The experiences of HCPs in our study confirm the view that wellbeing is multi-dimensional, but there were important differences in the nature of these dimensions when compared to other studies (Diener and Ryan, 2009; Linton et al., 2016; Ryan, 2009; Ryff, 2017). Ryff’s model of Psychological Wellbeing (2017) for example, is consistent with our findings on three dimensions, that is, purpose in life, positive relationships and environmental mastery (Ryff and Singer, 2008). However, Ryff’s three remaining dimensions of self-acceptance, autonomy, and personal growth, stand apart from our findings. The notable difference is that these three are self-focussed. This difference suggests that in a crisis, or a time of high stress, the focus on self, at least initially, diminishes in favour of focus on others and what one can do to help.

A CA is more nuanced than many other models, focussing as it does on individual agency *in relation* to a particular context. This subtle, yet important, difference allows greater scope for understanding the situational differences evident in a crisis situation. For example, HCP wellbeing was evident in a purpose-built ICU despite the extreme pressure and tight controls. Working in a rapidly assembled, makeshift ICU, however, was a completely different experience that seriously challenged HCP wellbeing (Ackley et al., 2024; Lee et al., 2020). Agency in this instance was not about personal freedom but about being able to perform well as a HCP in that particular situation. A CA reaches beyond the individual to assess how systems and social structures impact people endeavouring to fulfil their valued goals. As such it highlights the inequalities and disadvantages inherent in given social locations and systems. As Willis et al. (2021) point out Covid amplified the cracks that were already in the system.

It is clear, that wellbeing is not something one immediately associates with times of immense stress and pressure. It is more likely to be considered in relation to experiences of human flourishing, self-fulfilment, pleasure and welfare, and identified as a positive subjective experience marked by happiness, joy and contentment (Haybron, 2008; Ryan and Deci, 2001; Seligman, 2002; Seligman and Csikszentmihalyi, 2000). These things however seldom fit with HCP’s pandemic experiences. In our study being ‘happy’ was not the defining element. Instead, it was being able to realise one’s capabilities in achieving desired functioning. As Simons and Baldwin (2021) suggest, wellbeing in contrast to happiness, can potentially to a greater or lesser extent always be present.

Self-fulfilment is frequently referred to in wellbeing studies as being an important dimension of wellbeing (Abu Bakar and Mohamed Osman, 2021; Ivtzan et al., 2013; Taylor, 2011). Self-fulfilment does in some sense reflect capability but the HCPs who spoke of this tended to highlight the element of service to others, or contributing to one’s team, rather than themselves. That is, the capability had been transformed into a valued functioning that was the source of wellbeing itself. This recognition that capability underpinned valued functioning was also evident in China where Liu et al. (2021) found that frontline HCPs who had a stronger sense of work purpose and meaning exhibited higher levels of engagement and taking charge at work. This was consistent with our study, where illbeing arose from frustrated or negative functioning. Robeyns (2016) identified something similar in her study of autistic people who, when describing elements of wellbeing or illbeing, did not tend to talk about their preferences or desires but about what they were able to be and do in their lives.

### Study Strengths

A particular strength of this study was the inductive analysis of in-depth interviews. This meant that the identification of *capabilities* was not tied to an externally generated list but emerged from the HCPs themselves. This allowed greater insight into the inherent variability of HCP experience. Also, this study did not pre-define or pre-determine what constituted wellbeing but rather reflected on evidence that emerged spontaneously in interviews collected for a different purpose, providing a more nuanced understanding of wellbeing and how it featured in this crisis. Wellbeing was evident despite the situation and our aim was to understand more about this using a CA. A further strength was the international spread of participants which allowed some insight into the impacts of different governments and health systems on HCP wellbeing. It also highlighted the differential impacts resulting from different locations vis-à-vis the pandemic wave.

Using a CA allowed greater recognition of at least some of the multi-dimensionality and variability of workers’ situations, where serious challenges and deprivations were sometimes present at the same time as high levels of wellbeing in other areas. It was clear in this study that it would have been inappropriate to try and trade-off one capability against another in order to find a unitary value of wellbeing that could be used for comparison. Being high on one dimension does not necessarily compensate for being low on another. All dimensions need to be attended to (Robeyns, 2016).

Finally, a particular strength of this study is that it is one of relatively few that addresses wellbeing as a positive value rather than an absence represented in measures of distress. As a positive value it speaks more to HCP dedication and resilience.

### Study limitations

The qualitative design utilised in this study limits our ability to generalise beyond these data. Study participants were not a representative group and tended to be more experienced, with a number holding senior positions. There was a preponderance of Australasian respondents and insufficient representation from countries outside this region. This restricted our capacity to comment on the relative wellbeing impact, within and between different countries and health systems. It was clear however that the specific constraints impacting individual HCP’s *functionings*, differentially impacted their wellbeing. It can be surmised that this would also be a factor in staff resilience although this question was beyond the scope of this study. Further research is needed to explore these issues but the coherence that was apparent across our narratives, gives us confidence in our findings.

### Future research

Future research specifically designed to explore HCP wellbeing through a Capability lens, in conjunction with, or as an alternative to, the more common dysfunction (mental ill-health) lens, would benefit HCPs, institutions and health systems in their preparedness for future crises.

Research to better understand the link between HCP wellbeing and resilience could also influence workforce planning models and enable greater system resilience.

Comparative research exploring HCP wellbeing across different countries and health systems, would be of considerable value for understanding how health-system organisation impacts staff wellbeing.

This study also suggests that there would be considerable value in exploring a multi-disciplinary team approach to healthcare characterised by strong collegiality and reduced hierarchies. Utilising a CA to explore the potential of such teams would be useful.

The findings in this current study were specifically located in the early months of the pandemic. It would be valuable to do a Capability based analysis at the present time to understand more around wellbeing at baseline.

## Conclusion

The application of a CA in this research highlighted the importance of including individual situatedness as a significant factor for understanding wellbeing. It was evident HCP wellbeing was grounded in their sense of being supported (or not) in their work. However most of the wellbeing interventions rolled out for HCPs at this time, were not about strengthening this sense of support at work but were individually focussed aimed at enhancing each individual’s tool-kit for coping. There were lots of messages around diet, nutrition, sleep, managing stress and anxiety, practicing mindfulness, staying connected with others and so forth. These approaches are primarily based on the psychological models of wellbeing that predominate in western-style healthcare and healthcare management. The downside of this is that these models imply that wellbeing and coping is primarily, if not totally, the individual’s responsibility. The involvement of the workplace in staff wellbeing is at best understated and at worst hardly considered a factor at all. A CA questions the legitimacy of doing this.

Our research is suggestive of some key workplace adjustments that could greatly benefit HCPs and healthcare services going forward. Firstly, the value of promoting collegiality and a strong sense of ‘Team’ in the health workplace was very clear, especially teams that cross traditional or hierarchical boundaries. The sense of commitment and resilience this engendered among HCPs was very clear. Secondly having a pandemic or crisis plan in place and widely understood by the workforce before it was needed would help to facilitate the rapid adaptations required in order to meet the uncommon demands of crises. HCPs with previous crisis training and experience were an invaluable resource for those lucky enough to work with them. There was clearly a case for having a core team of specially trained HCPs available to lead the response across the sector.

## Supplemental Material

sj-docx-1-hea-10.1177_13634593241279206 – Supplemental material for Conceptualising wellbeing among health-care workers during the Covid-19 pandemic Supplemental material, sj-docx-1-hea-10.1177_13634593241279206 for Conceptualising wellbeing among health-care workers during the Covid-19 pandemic by Judith McHugh, Paul Trotman, Helen D. Nicholson and Kelby Smith-Han in Health
