# Supplementary material for: Conceptualising wellbeing among health-care workers during the Covid-19 pandemic
Source: Health (London). 2024 Oct 6;29(3):335–54. doi: 10.1177/13634593241279206 (PMC12049583; doi:10.1177/13634593241279206)
Supplement: sj-docx-1-hea-10.1177_13634593241279206 – Supplemental material for Conceptualising wellbeing among health-care workers during the Covid-19 pandemic [file sj-docx-1-hea-10.1177_13634593241279206.docx]

**Supplementary Material**

**Conceptualising wellbeing among health-care workers during the covid-19 pandemic**

## **Interview Guide**

The following questions were used to guide the interviews.

- Tell me when you first heard about the coronavirus and what you thought.
- When did you realize that this was serious?

Workplace Impacts

- How has this outbreak changed your practice?
- How have you prepared at work for this?
- What is the atmosphere like at work - with colleagues/employees, auxillary staff, suppliers etc?
- How has this affected the financial viability of your practice?

Working in PPE

- Have you spent much time in PPE? Do you use PPE at your practice?
- What was your access to PPE like?
- What's it like wearing PPE/working in PPE?
- How does wearing PPE affect your relationship with the patients?
- How do you smile in PPE?
- Would you still see patients if there was no PPE or what would you do if there was no PPE?

Dealing with uncertainty

- How are you coping with the uncertainty?
- How did you keep up with incredibly fast rate that the advice was changing?

Dealing with fear/anxiety

- What scares you about all this?
- Are you worried about catching the virus?
- It's not often that a healthcare practitioner goes to work and thinks I could die from my work. How do you cope with that?
- How do you prevent taking the virus home from work?

Sense of Self

- How has this changed how you see yourself as a healthcare practitioner?
- Is there anything you think you're doing particularly well?
- Is there anything that you think you're doing badly?

Has this changed how you see your patients?

- What have you Learned about yourself?
- What, what's the hardest thing you've had to do over this?

Other

- How well do you think the government has handled it?
- How do you see all of this playing out? How's it going to end?
- Is there anything else you want to talk about?
- What will you tell your grandkids about the coronavirus outbreak?
- Do you think the world will be the same again?

**Table 1: Thematic Analysis**

| **Codes** | **Categories** (associated code groupings) | **Concepts** (Ideas, notions & abstractions rather primary experience) | **Themes** |
| --- | --- | --- | --- |
| Physical Illness,  Anxiety, Fear,  Grief, Loss,  Depression,  Guilt,  Futility, Hopelessness, Helplessness, Failure, Anger, Confusion, Sadness, Self-doubt, Exhaustion,  Coping  Too much Information  Too much change | Vulnerability  Grief & Loss  Futility  Failure  Fear  Guilt Powerlessness  Overwhelm  Anger  Coping | Stress  Negative wellbeing  Distress  Coping | **Illbeing**  HCPs were confronted by significant, substantial and pervasive challenges to wellbeing in their work. |
| Team, camaraderie, collegiality, solidarity  Sharing, empathy, love  Helping & caring for each other.  Positive interactions  ‘Going extra mile.’ | Teamwork  Camaraderie  Trust  Empathy  Communication  Team management  ‘Everyone pulling together’ | Diminished hierarchies  Mutual support  Team  Responsiveness  Sense of connection  Collective Effort | **Wellbeing**  **Participating in positive Relationships** |
| Feeling   - Useful - Competent - Called - Challenged - Fulfilled - Putting self at risk to help - Faith in self/process/ others/ God | Sense of responsibility  Motivation  Service  Faith (in something) | Personal sense of meaning & purpose derived from professional role.  Job satisfaction  Sense of self  Self-awareness  Empowerment  Sense of Service | **Wellbeing**  **Sense of identity, purpose, meaning and value in relation to one’s work**. |
| Safety  Having knowledge & Skills  Training  Being able to help people.  Having a plan of action  Achieve good outcomes for others.  Decision making | Knowledge & skills  Effectiveness  Availability of resources  Planning  Patient outcomes  Decision making  Feeling supported  Good Leadership | Being Prepared  System support  Management  Co-operative decisions  Education & Training  Competence  Role performance  Leadership | **Wellbeing**  **Ability to provide an appropriate level of medical treatment, care, and other role-related support.** |
